# Experimental and Numerical Analysis of Mixed I-214 Poplar/*Pinus Sylvestris* Laminated Timber Subjected to Bending Loadings

**DOI:** 10.3390/ma13143134

**Published:** 2020-07-14

**Authors:** Francisco J. Rescalvo, Cristian Timbolmas, Rafael Bravo, Antolino Gallego

**Affiliations:** 1 Building Engineering School, University of Granada, 18071 Granada, Spain; antolino@ugr.es; 2 Department of Structural Mechanics, University of Granada, 18071 Granada, Spain; timbolmas@ugr.es (C.T.); rbravo@ugr.es (R.B.)

**Keywords:** poplar, pine, timber, glulam, bending, numerical modelling, non-destructive testing

## Abstract

The structural use of timber coming from fast growing and low-grade species such as poplar is one of the current challenges in the wood value chains, through the development of engineering products. In this work, a qualitative comparison of the behavior of mixed glued laminated timber made of pine in their outer layers and of poplar in their inner layers is shown and discussed. Single-species poplar and pine laminated timber have been used as control layouts. The investigation includes destructive four-point bending tests and three non-destructive methodologies: finite elements numerical model; semi-analytical model based on the Parallel Axes theorem and acoustic resonance testing. An excellent agreement between experimental and numerical results is obtained. Although few number of samples have been tested, the results indicate that the use of poplar as a low-grade species in the inner layers of the laminated timber can be a promising technology to decrease the weight of the timber maintaining the good mechanical properties of pine. Likewise, the need for the use of the shear modulus in both experimental measurements and numerical analysis is suggested, as well as the need to reformulate the vibration methodology for non-destructive grading in the case of mixed timber.

## 1. Introduction

The development of Engineered Wood Products (EWP) such as glued laminated timber or Glulam [1,2,3,4,5] enhance the use of wood with a wider range of benefits: (1) greater dimensional stability; (2) higher design flexibility, allowing timber to be produced in a wide variety of shapes from straight beams to curved arches. This fact offers a multitude of options for large and open spaces with a reduced number of columns; (3) cover longer span lengths and cross-sections, thus being a product of a huge variety of uses, from residential constructions up to commercial buildings and timber bridges where higher structural requirements are needed; (4) higher quality of the final product due to the grading of the planks, a selection according to their stiffness and mechanical properties and the removal of knots and another undesirable defects; (5) the use of a wood species with lower diameters when compared with sawn timber; (6) the use of lower-grade timber in lower-stress zones, resulting in a more efficient use and conservation of the timber resources. Regarding the last two benefits, poplar (*populus*) is one of the better candidates to be used for glulam timber. In Spain, poplar (*Populus x aeuroamericana (Dode) Guinier*) is one of the most important plantation species, covering an extension of 145,000 ha approximately according to FAO [6,7]. Currently the use of poplar is mainly used for peeling and plywood manufacturing. The development of EWPs and its use for structural purposes is a huge opportunity to develop all the wood value chain.

Combination of different wood species has a wide range of structural advantages being introduced by Biblis [8]. In [9], the authors developed a theoretical analysis and performed an evaluation by means of twenty large beams graded visually and made of Douglas-fir (*Pseudotsuga menziesii*) and lodgepole pine (*Pinus contorta*). Results demonstrate that the low-grade wood, i.e., lodgepole, with similar mechanical properties than poplar, used in the inner part of the glulam beams, had a little effect on the general mechanical properties. Previous works studied the mechanical properties of poplar glulam beams [10,11]. In particular, Martins and collaborators used this grade consideration to sort the plank for every glulam beam by using the longitudinal vibration method (LVM) [11]. That work correlates the non-destructive testing with the mechanical properties of poplar glulam beams considering 4 types of adhesives. Authors also compare the results with the transform section method (TSM) calculated by using the modulus of elasticity of each plank. Results demonstrate a very good performance between TSM method and the global modulus of elasticity, with a correlation coefficient of 0.93, and a good correlation of 0.8 between LVM method and the global modulus of elasticity. A comparison between single-species glulam poplar (*Neva* clone, with a low density) and eucalyptus beams and mixed beams by using four types of eucalyptus clones was performed by Castro et al. [12]. For the grading of the planks, they performed a quasi-nondestructive static bending test applying low loads to them. Mixed beams designs consisted in five inner poplar planks and two outer eucalyptus planks. In general terms, results demonstrate that the combination of species and the use of the low-grade species in the inner part of the glulam timber enhance the modulus of elasticity between 9% and 51% depending on the eucalyptus clone. To evaluate the ductile behavior of mixed glued laminated timber, the authors of [13] also considered poplar for the inner part of the beams, mainly due to its low density and good shear performance, using Norway spruce and larch wood for the outer planks. In addition, authors evaluated the mechanical behavior elaborating glulam timber from the same wooden species but different strength classes (as Hernandez and collaborators proposed for poplar glulam timber [14]). Results demonstrate that combination of species results more efficient when they have clearly different strength limits between them. Authors also remarked the high importance of an accurately grading of the planks during the design process. In [15,16,17], the authors also combined different species of wood with the same final conclusion of low-grade, high-grade timber distribution for inner and outer parts of the glulam timber, respectively. Furthermore, this conclusion has been recently extended to CLT (Cross Laminated Timber) panels [18]. In [16], when a high-grade wood such as Merpauh was used for the outer planks, improvements of the modulus of elasticity between 117% and 157% were achieved.

An improvement method in [19] was presented by Shin et al., taking into account the neutral axis shifts in bending using the Time-of-Flight method. Authors took into account the differences between compression and tensile modulus of elasticity, improving the relationship between static and dynamic elastic moduli. Despite taking into account the neutral fiber axis shifts, results from previous works [20,21] for sawn timber, demonstrate that resonance method results in a more reliable technique to obtain the dynamic modulus of elasticity, since this method consider the whole piece of wood. Finite Element Method is powerful tool to predict the elastoplastic behavior of natural beams with flaws, as knots and grain deviation in bending [22]. It can reproduce with a good accuracy the four-point bending tests laminated beams (see, e.g., the application of FEM (Finite Element Method) to Cathay poplar glulam beams in [23]).

In order to qualitatively assess the behavior of a mixed glulam timber using poplar wood for the inner part and pine for the outer layers, three layouts have been tested in this work: (1) single-species pine timber used as control specimens; (2) single-species poplar timber used as control specimens; (3) mixed poplar/pine timber. To ensure a good performance and a proper sample design, all the planks were graded individually by means of the resonance acoustic testing. Subsequently, the samples were subjected to a four-point bending test following the standard [24], and the results were compared with three non-destructive methodologies: (1) numerical finite elements model (FEM); (2) Parallel Axes theorem; (3) acoustic resonance testing (ART). All the samples were graded and compared according the standard and an analysis of the effectiveness of the use of poplar for the inner layers has been carried out. Due to the low number of samples used for each timber layout, the results of this work should be considered only in qualitative and not in quantitative terms. In any case, since the main objective of this work is to compare the proposed numerical method with the experiments, the results make it clear that after calibration and using the shear modulus in the formulation, an excellent agreement is obtained between numerical and experimental results.

## 2. Materials and Methods

### 2.1. Experimental Program Flow

**Figure 1:**
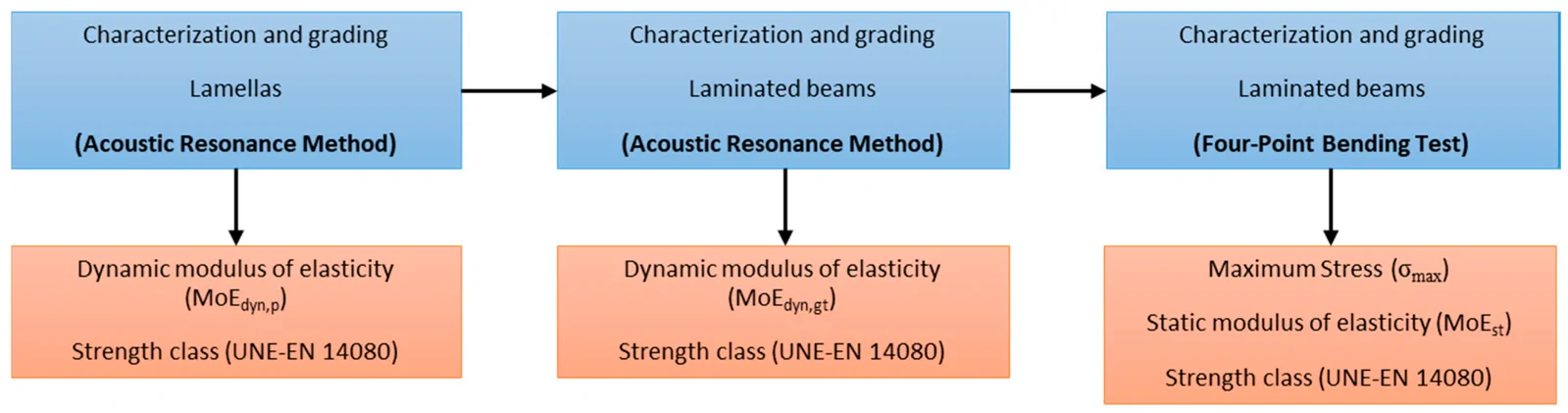
Chart flow of the general experimental procedure. MoE_dyn,p_: Dynamic modulus of elasticity of planks. MoE_dyn,gt_: Dynamic modulus of elasticity of glulam timber. σmax: Maximum stress in bending. MoE_st_: Static modulus of elasticity.

The experimental program followed the chart flow shown in Figure 1. Poplar and pine planks used for the glulam timber samples were graded according with their dynamic modulus of elasticidity, MoE_dyn,p_, obtained by Acoustic Resonance Testing (ART). Based on this grading, design and then manufacturing of the laminated timber were carried out. After that, timber was subjected to ART in order to obtain their dynamic modulus of elasticity, MoE_dyn,gt_. Finally, they were destructively tested in bending, thus obtaining their static modulus MoE_st_ and the maximum stress in bending, σ_max_.

### 2.2. Raw Material and Planks

Two species were used, *Pinus sylvestris* and poplar clone I-214 (*Populus x aeuroamericana (Dode) Guinier*). Thirty-four pine planks were extracted from the same sawn timber batch at 90 years old forests of Soria province (Spain), supplied by Madera Pinosoria S.L. Similarly, 14 poplar planks were extracted from the same sawn timber batch from a 13 years old poplar plantation located at Yunquera de Henares (Guadalajara, Spain). Both pine and poplar timber were artificially dried, ensuring a final moisture content (MC) of 12%. Planks had a cross-section of 20 × 50 mm^2^ and a total length of 1240 mm. The mean density at MC = 12% for the pine and poplar planks was of 526 ± 55 and 347.3 ± 22.7 g/cm^3^, respectively.

### 2.3. Dynamic Modulus of Elasticity of Planks: Acoustic Resonance Testing

**Figure 2:**
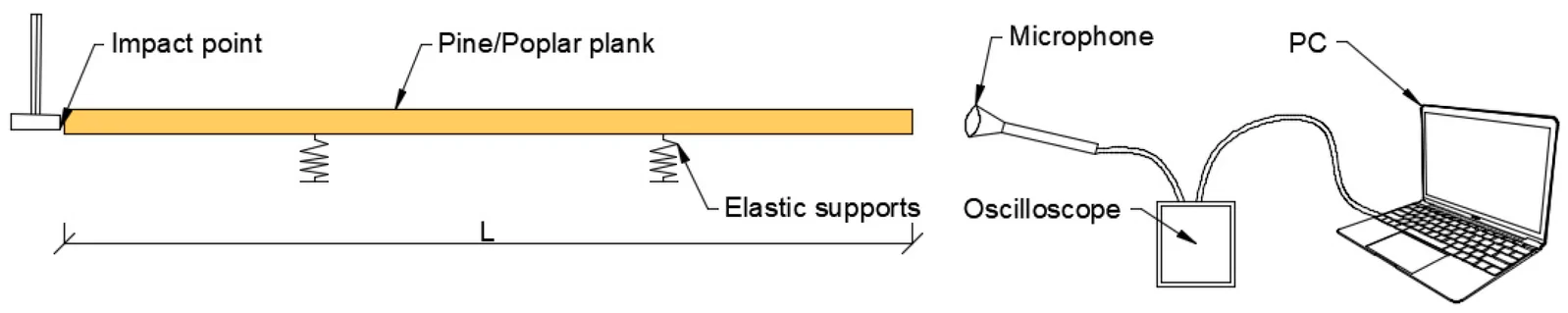
ART layout for planks. L = Plank length.

All the planks were subjected to an ART [20,21] in a flatwise orientation by placing them on two elastic supports and hitting them with a hammer (Figure 2). Response vibration signals along the longitudinal direction were collected with a t.bonne MM-1 Thomann microphone (Thomman GmbH, Burgebrach, Germany) and transduced to an electrical signal recorded with a Picoscope^®^ 4424 oscilloscope with 80 MS/s (Pico Technology, Cambridgeshire, UK). The fundamental resonance frequency f_1_ of each plank was obtained by means of spectral analysis. Using this frequency and the density of each plank (ρ_p_), the dynamic elastic modulus (MoE_dyn,p_) can be estimated as follows:

(1) $$v=2{\mathrm{Lf}}_{1}$$

(2) $${\mathrm{MoE}}_{\mathrm{dyn},p}^{*}={\;\rho }_{p}v^{2}$$

where L is the plank length, and v is the propagation velocity of the stationary elastic wave. Furthermore, a correction of the dynamic elastic modulus (MoE_dyn,p,12_) from the real MC_p_ measured with a digital moisture meter to the MC = 12% was carried out according to the standard [25] as

(3) $${\mathrm{MoE}}_{\mathrm{dyn},p}={\mathrm{MoE}}_{\mathrm{dyn},p}^{*}\cdot \left(1+0.01\left({\mathrm{MC}}_{p}-{\mathrm{MC}}_{12\%}\right)\right)$$

**Figure 3:**
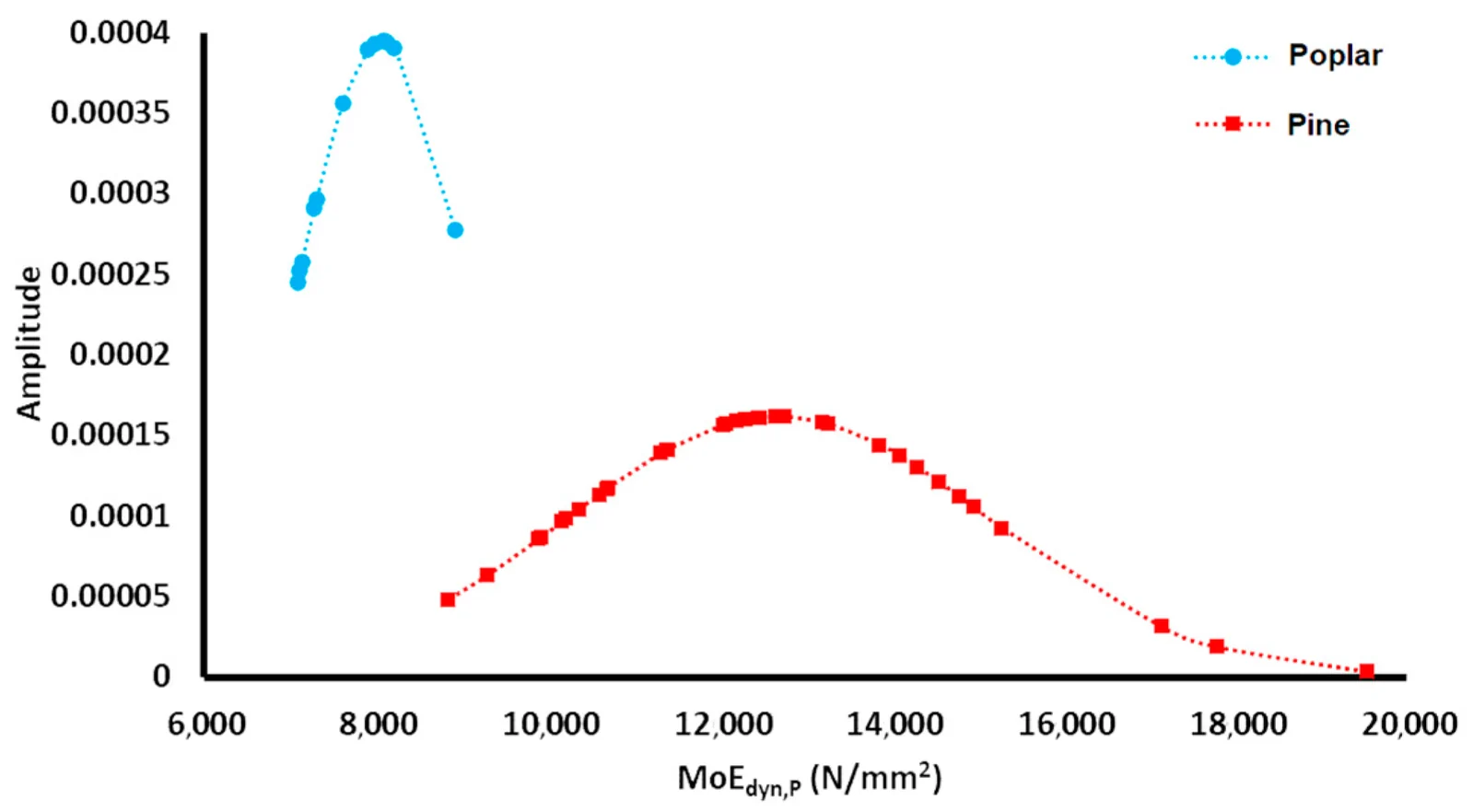
Normal distribution of the dynamic modulus of elasticity of the planks.

Figure 3 depicts the dynamic modulus of elasticity of all the planks. Results for poplar and pine follow normal distributions, with smaller values of the dynamic elastic modulus and a more reduced deviation values for the case of poplar timber compared with pine.

### 2.4. Planks Strength Grading

**Figure 4:**
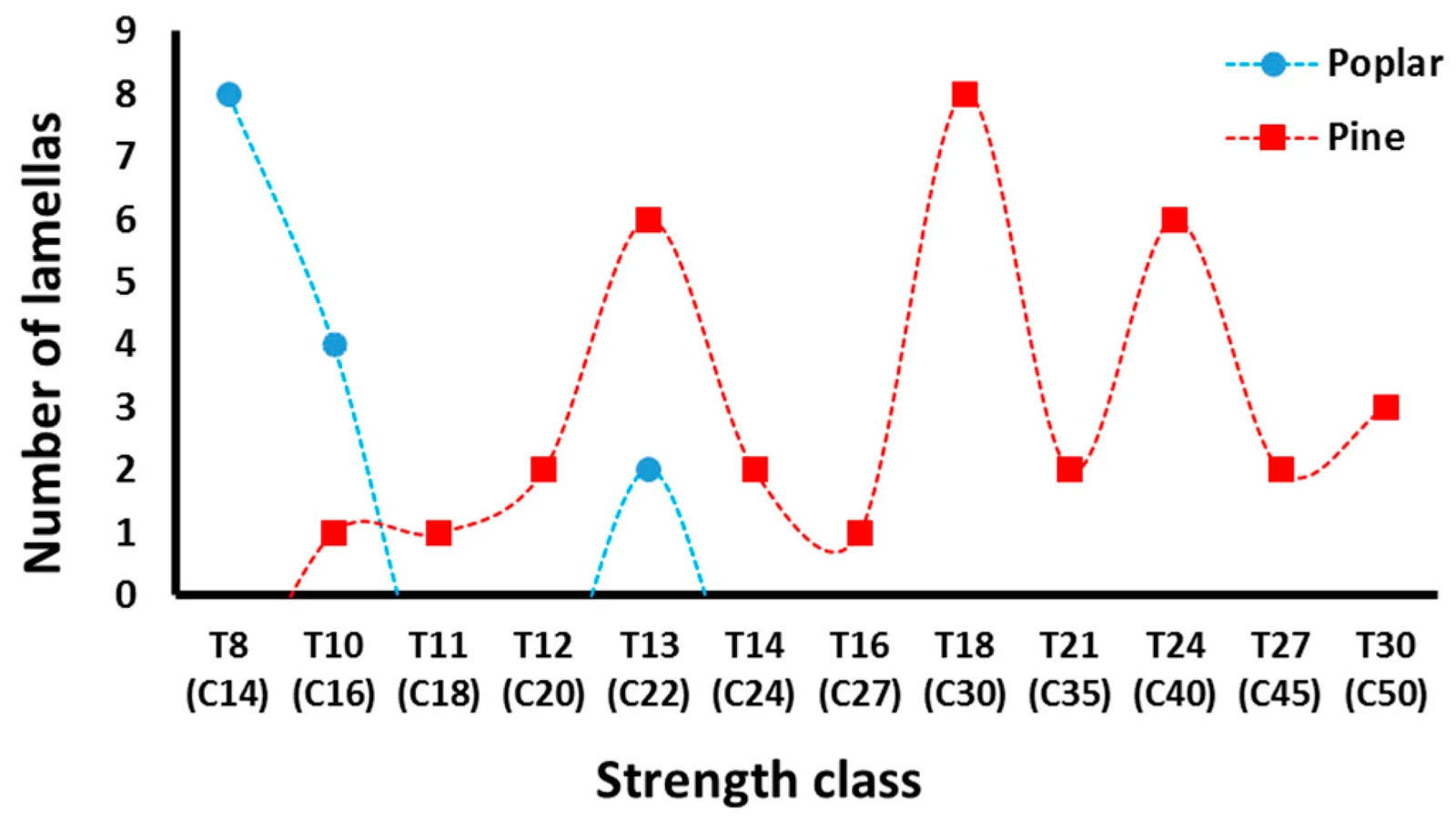
Distribution of planks according with the T and C strength class.

By using the MoE_dyn,p_, all the planks were graded according to Table 1 in Section 5.1.4.1 from reference [26]. That standard also associates the T strength class of the planks, which comply with the minimum values of C strength class for sawn structural timber according to standard [27]. Figure 4 shows the distribution of planks for each particular T and C class. Within each T strength class, the planks were considered structurally equivalent and were randomly selected for glulam timber manufacturing.

### 2.5. Glulam Timber Samples

**Figure 5:**
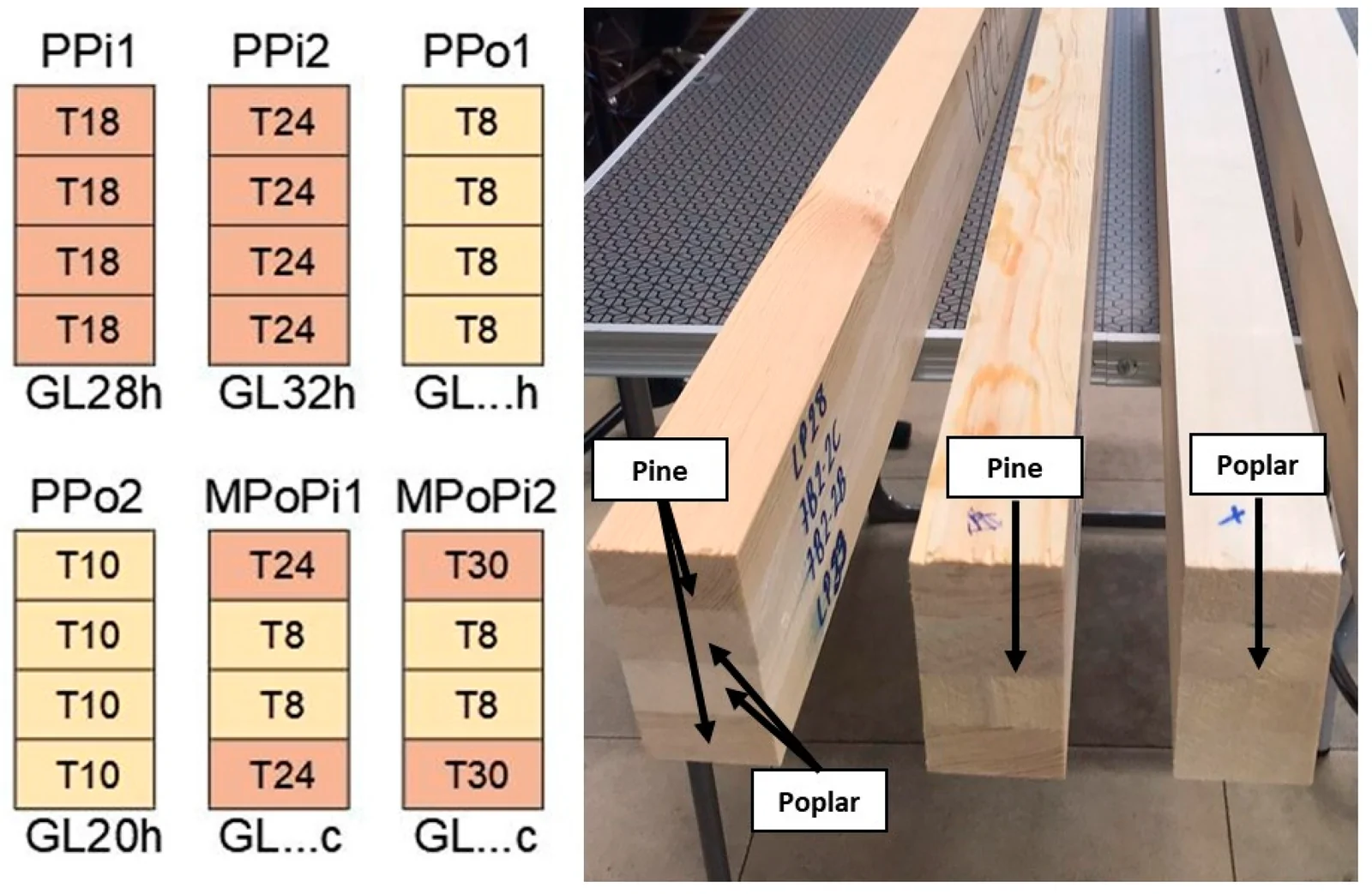
Timber layers design according with planks grading and strength class GL proposed by the standard [26]. PPi: single-species pine. PPo: single-species poplar. MPoPi: mixed poplar/pine. GLXc: design grading for combined. GLXh: design grading for homogeneous.

Three different types of 4-layers laminated timber were designed and compared; single-species poplar (PPo), single-species pine (PPi) and mixed poplar/pine (MPoPi) as shown in Figure 5. Two samples were manufactured for each type. Based on the T strength class of the planks and the recommendations for homogeneous timber given by Table 2 in Section 5.1.4.3 from reference [26], different layouts were set up for the case of pine or poplar single-species layouts used as control specimens. Figure 5 indicates also the GL strength class theoretically assigned by the mentioned standard (named in this paper as “design class”), except for the case of poplar/pine mixed timber because the standard does not consider multispecies layouts, and for the PPo1 sample due to the very low out-standard strength class of the poplar planks (T8). It should be also noticed that the pine planks of the single-species pine control sample PPi2 and mixed sample MPoPi1 have the same design class—T24. Thus, its mutual comparison can be used to evaluate the influence of the substitution of inner pine planks by poplar ones.

The planks were glued to each other by using the polyurethane resin PUR-20 from Bakar^®^ (Bakar, Vizcaya, Spain) with an amount of adhesive of 350 g/m^2^, applying a constant pressure of 6 × 10^−7^ MPa during 4 h without using any finger joints. The elaboration process was carried out following the requirements of the standard [26]-(Annex I). During the elaboration process the temperature and humidity of the room (HR) was of 20 °C and 40%. As indicated by the standard, the time between mechanization and gluing of the planks was lower than 24 h. The resulting dimensions of the samples were b = 40 mm, h = 80 mm, and L = 1230 mm.

### 2.6. Dynamic Modulus of Elasticity of the Glulam Timber

Each particular sample was subjected to a longitudinal ART [20,21] as described in Section 2.3 and shown in Figure 2, thus obtaining the longitudinal dynamic modulus of elasticity (MoE_dyn,gt_), including the MC = 12% correction for each sample.

### 2.7. Semi-Analytical Modulus of Elasticity

Since the samples are formed by planks with similar elastic properties, the Parallel Axes theorem [28] can be used to semi-analytically obtain a combined modulus of elasticity, MoE_c_. It was calculated by using the experimental dynamic modulus of elasticity (MoE_dyn,p_) for each individual planks, as

(4) $${\mathrm{MoE}}_{c}=\overset{N}{\underset{l=1}{\sum }}\frac{{\mathrm{MoE}}_{\mathrm{dyn},p}\cdot I_{p}+A_{p}\cdot {\mathrm{MoE}}_{\mathrm{dyn},p}\cdot y_{p}^{2}}{I_{c}}$$

where Ip is the second moment of inertia respect to sample axis, Ap is the cross-section of the sample, y_p_ is the distance from the combined neutral axis to the neutral axis for each particular plank p, and I_c_ is the combined second moment of inertia.

### 2.8. Bending Test

**Figure 6:**
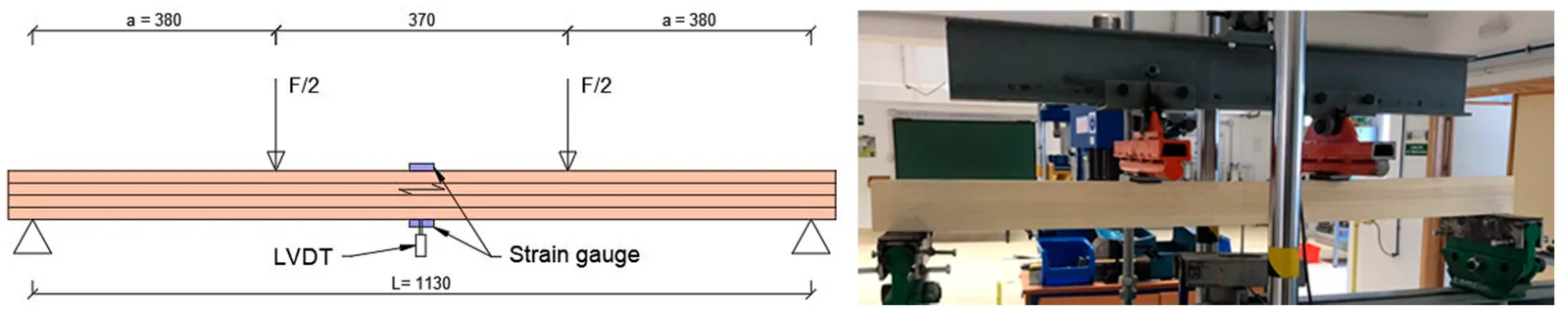
Left: Four-point bending test arrangement. Right: single-species poplar sample during the bending test. Distances in mm.

**Figure 7:**
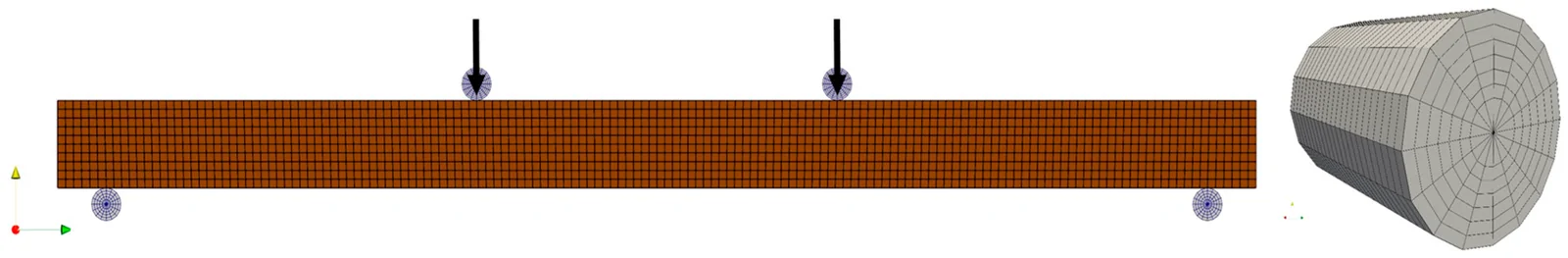
Left: 3D-FEM model for a four-point bending test set-up. Right: 3D-Mesh of load cells/supports.

Following the standard [24], a monotonic four-point bending test was performed for each particular sample (see Figure 6). Due to the plank sizes and in order to avoid torsion effects caused by a small base of the samples, a 14h ratio was set, scaling the standard arrangement. Displacement control ratio was set at 4 mm/min, in order to fulfill the requirements of the aforementioned standard. A 100 kN-capacity testing machine (Equipos de ensayo Controls S.A., Toledo, Spain) was used. Two strain gauges were glued at the mid-spam section of the sample on the bottom and top faces, in order to measure the maximum tensile and compression strains, respectively. The span between supports was set as 1130 mm. The maximum stress σ_max_ was calculated as

(5) $$\sigma_{\max}=\frac{M_{\;\max}}{W}$$

where M_max_ = F_max_ a/2 is the maximum bending moment and W is the section modulus (see Figure 6 for the meaning of a). Similarly, the static modulus of elasticity MoE_st_ was calculated by using the stress-tensile strain curve, as the slope in the linear range between 20–40% of the maximum stress. Moreover, the global modulus of elasticity MoE_st,g_ was also obtained according to Section 10.3 from reference [24], as

(6) $${\mathrm{MoE}}_{\mathrm{st},g}=\frac{3{\mathrm{aL}}^{2}-3a^{3}}{2{\mathrm{bh}}^{3\left(2\frac{\delta 40-\delta 20}{L40-L20}-\frac{6a}{5\mathrm{Gbh}}\right)}}$$

by using the load–displacement (L–  δ ) curve, in the same load range as MoE_st_ (20–40% of the maximum load). The displacement was measured by means of a LVDT placed as shown in Figure 7.

### 2.9. Strength Grading of Glulam Timber Samples

All the mechanical properties were considered initially for the strength grading but the density and maximum stress in bending were not the critical properties for strength grading. Thus, all the samples were graded taking into account the critical property, the modulus of elasticity as follows: (1) Using the dynamic modulus MoE_dyn,gt_; (2) Using the semi-analytical combined elastic modulus MoE_dyn,c_; (3) Using the static modulus MoE_st_.

### 2.10. Numerical Modelling

A three-dimensional finite element model (3D-FEM) was developed into the open source finite element code Salome Meca^®^ (Version 2019, Code Aster, France) [29] to evaluate the behavior of the manufactured glulam timber subjected to four-point bending test. The samples were modeled as elasto-plastic solids and nonlinear analyses were performed using a linear elasto-plastic constitutive model with hardening. The constitutive model received as input parameters the elastic modulus, shear modulus and Poisson ratio for the linear elastic part, and the yield stresses for the plastic part with hardening. The 3D-FEM model consists of one piece with a rectangular cross-section and four simply supported solid rollers (see Figure 7). Bottom rollers represent the supports and top ones the points of application of the loads in the testing machine. In order to get accurate results with reasonable computational cost, an analysis of convergence of results dependent of element size was carried out. An optimum size of 8 mm was achieved and the sample was meshed into 9240 eight-node brick elements having a total number of 11,935 nodes. Each roller was meshed into 1648 six-node wedge elements with a total number of 1109 of nodes, allowing a proper adaptation to the cylindrical shape. The right subfigure in Figure 7 shows the defined mesh for the supports and load cells in detail. The average radial size for each element was 2.75 mm and an outermost size of 5.85 mm was adopted, resulting in a total roller diameter of 30 mm. Due to the set-up of the experimental part, four-point bending test, the material definition of the 3D-FEM model was considered to be isotropic. In this case, the specimen is working mainly in the longitudinal direction without any transverse constraints and the mechanical properties in the longitudinal direction are the most relevant for this analysis. The isotropic model also implies an intermediate-low computational cost without important influence in the results. Rollers for the experimental test were made of steel so, in consequence, the elastic modulus of 210 GPa and the Poisson’s ratio of 0.3 were considered.

In coherence with the experimental test, bottom rollers were restrained to move in any direction and the top rollers (points of load application) were constrained to remain horizontal, while being able to rotate and moving vertically during the simulation. Previous authors [23] use similar numerical setups without modelling contact interactions from supports and loads cells. Further, a contact restriction between roller and the laminated specimen surface was prescribed. Definition of contact requires the description contact candidates which comprise the slave and master surfaces [30]. The contact formulation, applied to the simulation, are normal and frictional penalties [31] due to their ability to soften the nonlinearities induced by contact. The mechanical stiffness of penalty springs was calibrated to 1 × 10^9^ kN/m according to the guidelines given in [32], while the friction coefficient was set to 0.3, which is a common value for the friction for steel surfaces. In order to simulate the whole experimental process and to improve the convergence of the contacts, the load increases linearly and is applied in the center of gravity of each top roller.

The set of nonlinear equations from the finite element model was solved with a direct full Newton nonlinear solver. The solution provides the displacements, elastic/plastic stresses and strains at each increment. A set of nodes located at the bottom face of the mid-span, in the same positions as LVDTs, was used to obtain the deflection during the simulation.

Moreover, in order to obtain the mechanical properties for the FEM model, a calibration process was carried out. It consisted of running the analysis using a starting value of elastic modulus, equal to the one determined through the experimental part (MoE_st_), and then the elastic and plastic parameters as the yielding stress (σ_y_) were adjusted to fit the experimental patterns. In order to compute a reasonable shear modulus (G), MoE_st_ is enforced to be equal to MoE_st,g_ in Equation (6). The rest of known parameters from Equation (6) represent: L, the span of the sample; a, the distance between the supports and its nearest point of load application; b, the base of the sample; h, the height of the sample; L20 and L40, the load at 20% and 40% of the maximum load, respectively; and δ 20 and δ 40, the corresponding displacement registered with de LVDT at that load values. Thus, the only unknown to be solved is the value of the shear modulus G. The Poisson’s ratio (ν) was set as 0.37 by means of tests performed in [33]. Two calibration processes were carried out by considering G or not, thus obtaining the calibrated modulus of elasticity MoE_FEM,G_ and MoE_FEM_, respectively. In both cases, the calibration procedure was considered to be finished when the difference between numerical and experimental stiffness in the elastic regime was lower than 5%. Increments of applied load,  ΔL , and corresponding displacements,  Δδ , were used to compute the stiffness K applying the following relation:

(7) $$K=\frac{\Delta L}{\Delta \delta }$$

Stiffness of the samples was computed between 15 and 35% of the elastic range, based on the force–displacement relationship, for both the numerical and experimental part.

## 3. Results

**Figure 8:**
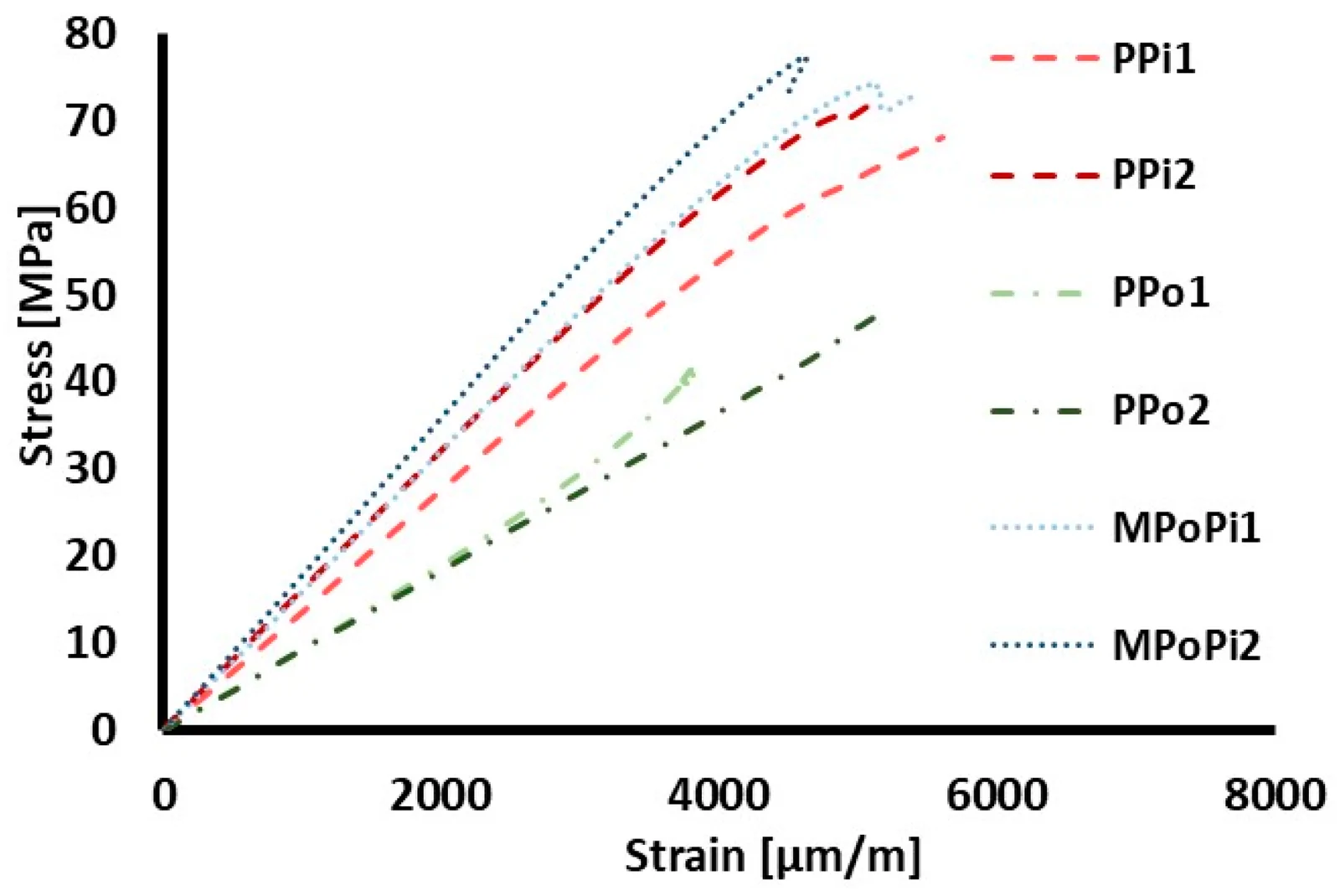
Stress versus strain for the tensile strain gauge (bottom face) for all the samples.

**Figure 9:**
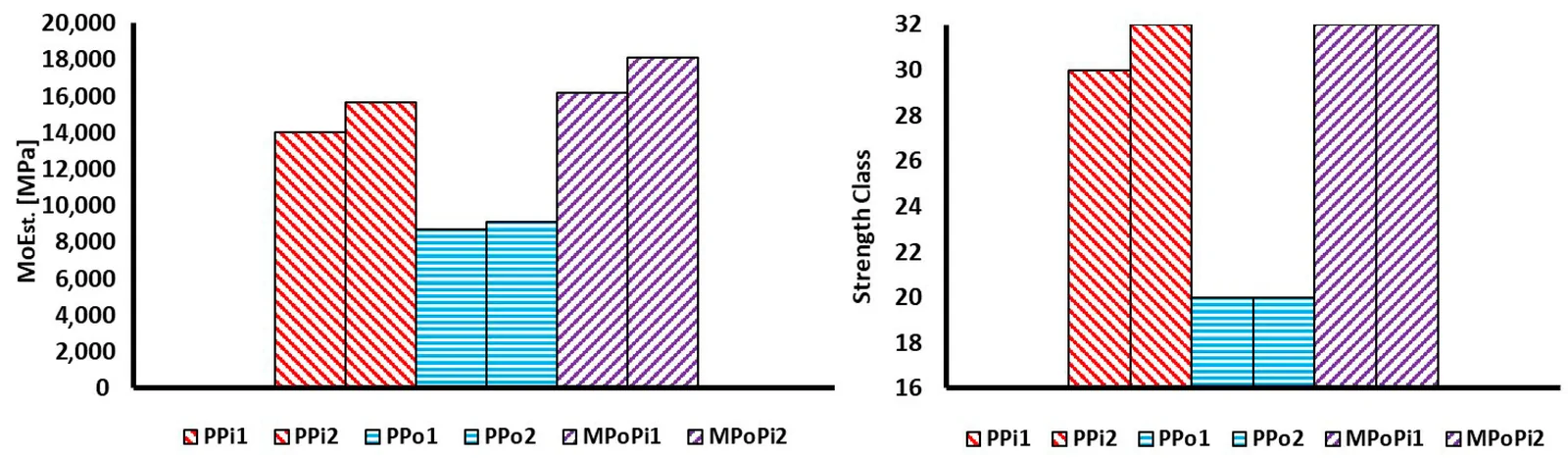
General results for the tested samples. Left: Static modulus of elasticity (MoE_st_). Right: Strength grading of the samples with MoE_st_.

**Figure 10:**
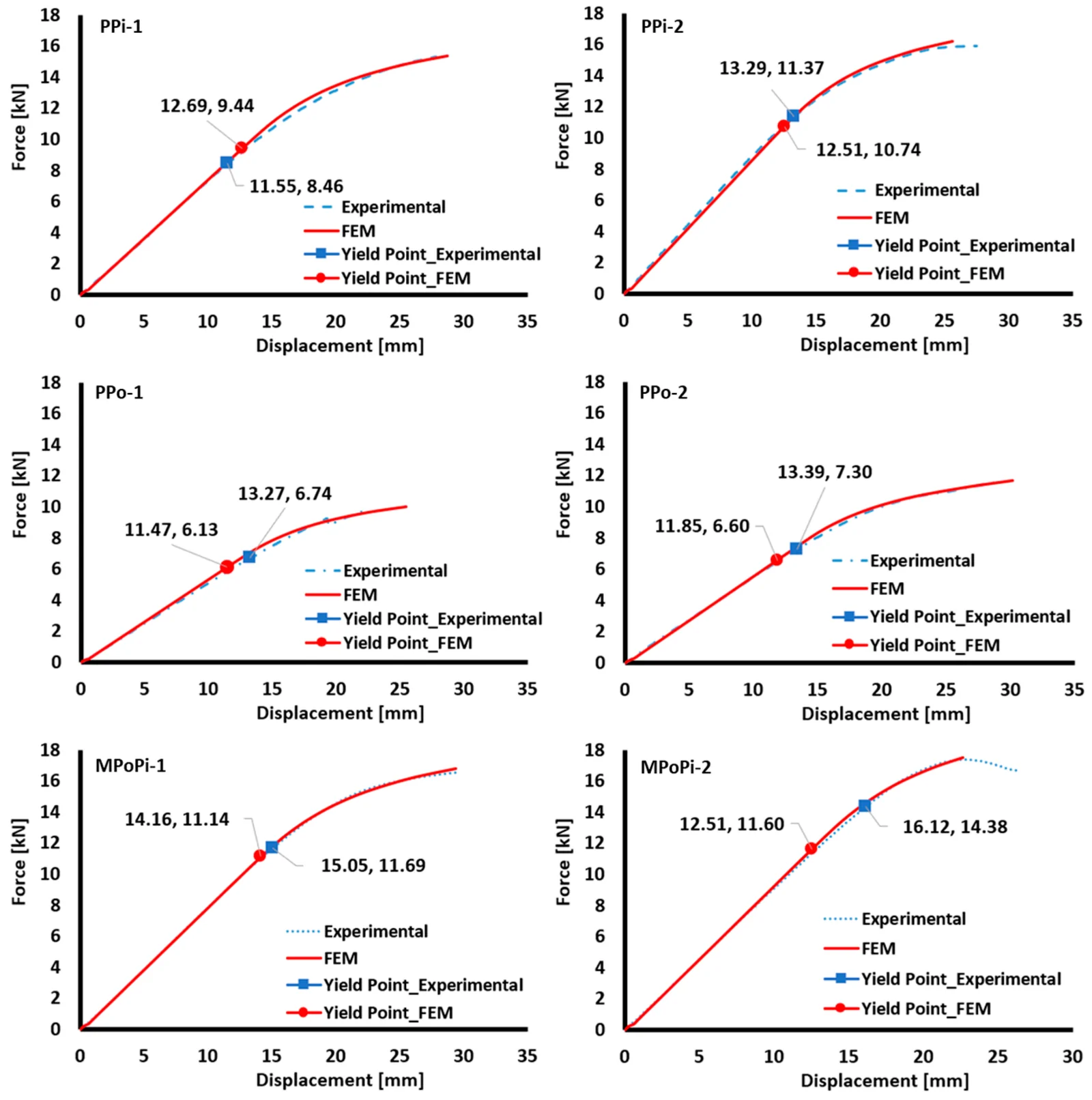
Numerical and experimental load-displacement plots and their corresponding yield limits.

**Figure 11:**
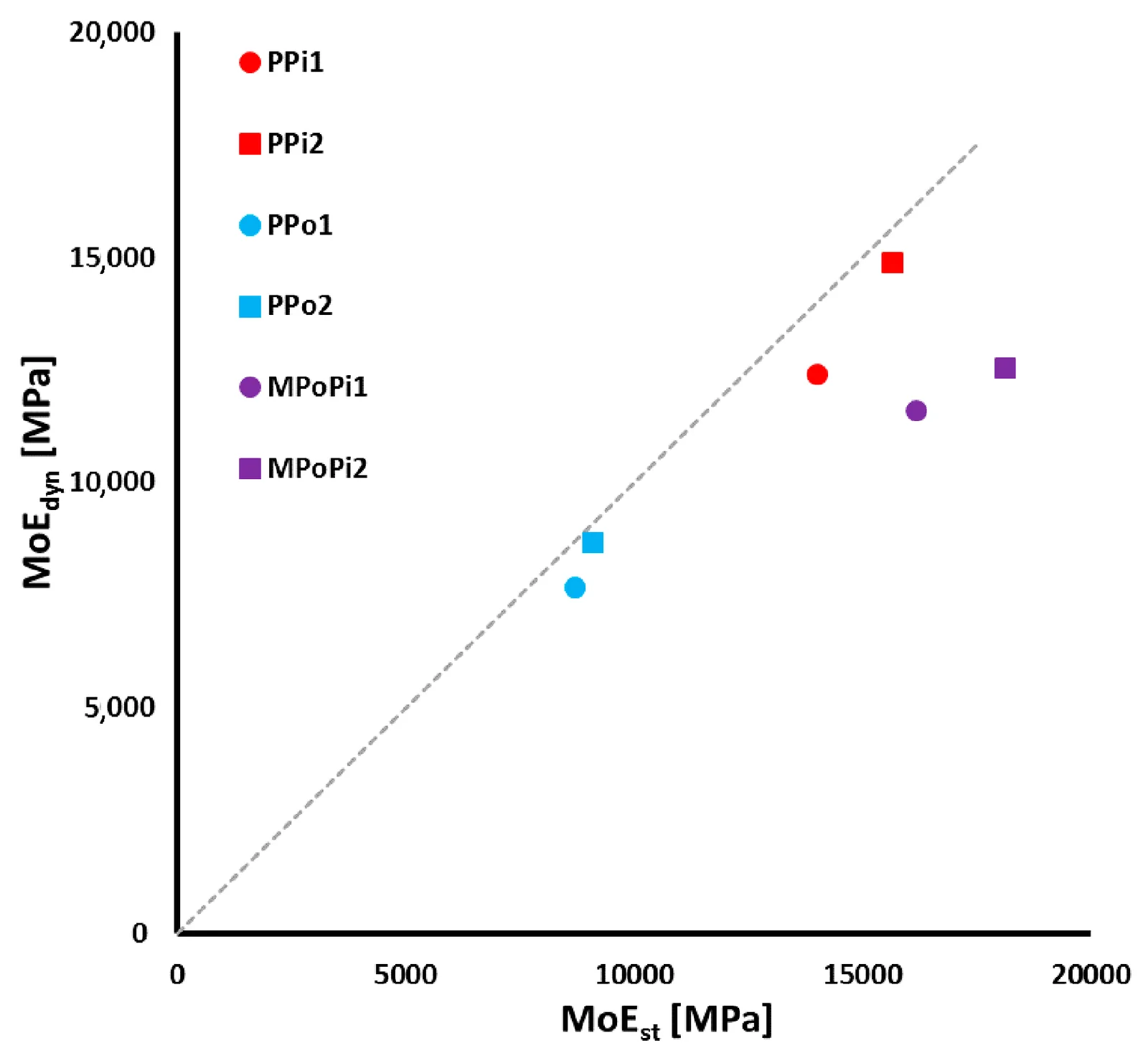
Comparison between modulus of elasticity for the tested samples. MoE_dyn,gt_: dynamic modulus of elasticity. MoE_st_: static modulus of elasticity. Gray dotted line: y = x.

Figure 8 shows the stress-strain curves for all the samples by using the tensile strain gauge. The samples are clearly grouped in three groups, following the order MPoPi > PPi > PPo. The single-species pine PPi2 sample and mixed MPoPi1 sample had very similar behavior, as Figure 8 shows. This relationship can be also observed in Figure 9, which compares the different static modulus of elasticity and strength class using the MoE_st_. Figure 10 plots the load-displacement relations and their corresponding yield limits, comparing experimental and numerical results. An excellent agreement can be observed for all the tested samples. Table 1 summarizes the results obtained for each sample, i.e., static, dynamic, semi-analytical, and numerical modulus as well as the maximum stress. The four parameters also follow the order previously mentioned, i.e., MPoPi > PPi > PPo. Figure 11 represents the relationship between the static and dynamic moduli, in which it can be observed that the higher variations were reached by the mixed samples. Table 2 presents the strength grading results with the different elastic moduli.

## 4. Discussion

It can be observed that, when the design strength class is available, a good agreement with the experimental and design strength is achieved. Moreover, as expected, higher mechanical properties are obtained for single species pine and mixed poplar/pine samples, compared with those for single-species poplar samples, due to the lower grading of poplar timber compared with pine. However, similar values of MoE_st_ are obtained for single-species pine sample PPi2 and mixed sample MPoPi1, the difference being no bigger than 3%, since they both used T24 pine planks in the outer layers. This demonstrates that the inner layers do not contribute significantly to the sample elastic modulus, allowing the use of low-grade species such as poplar for these inner planks. As mentioned by Moody [9], when mixing species, the outer layers of the samples provide the main stiffness contribution to the total sample. In particular, it is observed that by applying the Parallel Axes theorem (Equation (4)) and using the dynamic elastic modulus obtained for each plank, it is possible to obtain the contribution of each particular plank to the total modulus. As an example, Table 3 presents the results for the single-species pine PPi2 and mixed poplar/pine MPoPi1 samples, with their corresponding planks strength classes and dynamic moduli of elasticity. The results clearly demonstrate that for both planks the outer planks contribute with 87% and 95% of the total stiffness for the PPi2 and MPoPi1 planks, respectively, just leaving only 13% and 7% of modulus contribution for the inner planks.

Table 4 shows the variations of the numerical, dynamical and semi-analytical moduli respect to the static modulus MoE_st_. It can be observed that the variations between experimental and numerical results are very small, always below 8% for all the types of samples. In many cases, those differences are even less than 2%, demonstrating the effectiveness of the numerical modelling and calibration procedure using the shear modulus. More in detail, as shown in Figure 10, an excellent performance is achieved in the elastic range. Meanwhile, when plastification occurs, both experimental and numerical curves start to diverge, mainly due to the complex phenomena at this load stage (cracking, debonding, etc.). Moreover, the limit between elastic and plastic ranges is clearly separated by the definition of the yielding points for both approaches, as shown on the Figure 10. A big similarity is observed for this transition for both experimental and numerical methodologies.

When comparing the dynamic modulus and the semi-analytical moduli with the static modulus MoE_st_, the variations become lower than 15% for the case of pine and poplar single-species samples, demonstrating the effectiveness of these non-destructive methodologies. It can be observed how the results (elastic modulus and strength grading) are very similar to each other with both non-destructive methods. However, big variations are observed for the case of mixed samples, around 40% for the case of the dynamic modulus and 20% for the semi-analytical one. These high variations can be associated to the heterogeneity of the cross-section and the high variation of the strength class of the planks, T8 for the inner layers and T24 and T30 for the outer layers, respectively. Considering this fact, both non-destructive methods (especially the ART carried out on the whole laminated sample) are unable to provide reliable strength grading. These results are confirmed in Figure 11, in which the points corresponding to the mixed samples significantly move away from the y = x straight line. However, the points corresponding the single-species samples fall almost on this straight line.

It should be emphasized that the good agreement between numerical and experimental moduli is mainly due to the calibration process using the shear modulus (G). In order to discuss this issue, the influence of the shear modulus (G) is analyzed in Table 5, in which the experimental global modulus MoE_st,g_ and the numerical modulus MoE_FEM_, and their variations respect to the static modulus MoE_st_, are shown. The global modulus MoE_st,g_ is obtained experimentally, and it does not include the shear effect. Similarly, MoE_FEM_ is calculated without including G in the model. It is clearly observed that in both cases, very high variations are obtained (between 9–50%). It demonstrates the need to include G in both experimental global modulus and numerical modelling procedures. As a reference, Table 6 shows the value of the experimental and the calibrated shear moduli. A big similarity is obtained between them for all the tested samples.

Finally, Table 7 depicts the stiffness obtained from the numerical simulations and experimental data from Figure 10. As mentioned above, the elastic range is very similar for both phases, supported by a low value of error between numerical and experimental stiffness, which is below 4%. It is clear that the single-species poplar samples show the lowest stiffness as confirmed by the results, while the mixed pine poplar sample MPoPi2 show the highest one, due to the existence of the T30 plank’s strength class at the outer layers. The single-species pine samples show a relatively small reduction in stiffness compared with the MPoPi-2 specimen, which demonstrates that the extreme fibers have the highest contribution to the general strength of the sample. In particular, by comparing PPi2 and MPoPi1 samples, which are composed by the same strength class of outer planks, T24, it is clear that the insertion of poplar planks at the inner part produces a very small reduction in stiffness. Similarly, the results from Table 7 also show that the shear modulus does not play an important role in the computation of stiffness (K), keeping a variation below 3.5% with and without using G.

## 5. Conclusions

An experimental and numerical comparison of the mechanical behavior and strength grading between single-species pine and poplar and mixed poplar/pine laminated timber has been carried out. The main conclusions of the paper are as follows:

- Higher mechanical properties are obtained for single-species pine and mixed poplar/pine laminated timber, compared with single-species poplar specimens, due to the lower grading of poplar timber compared with pine.
- The inner planks do not contribute significantly to the whole sample elastic modulus, allowing the use of low-graded species such as poplar for the inner layers of the sample.
- After calibration and using the shear modulus in the formulation, a good agreement is obtained between numerical and experimental results. The use of the shear modulus in the formulation must be considered in order to obtain very low variations with the experimental results. Similarly, the experimental global modulus—without including the shear effect—does not provide reliable results with very high variations.
- The dynamic modulus and the semi-analytical elastic modulus obtained from the ART non-destructive grading of the whole sample or particular planks, respectively, is in good agreement with the static modulus and consequent strength grading for the case of single-species samples, with relatively low variations.
- Non-destructive testing grading based on ART provide a very low grading yield for the case of mixed samples, with very high variations. Thus, this methodology should be reformulated and adapted in the future in order to get more reliable grading results.
- The insertion of low-grade poplar planks at the inner layers produces a very small reduction in the stiffness compared with the single-species pine type.
- The shear modulus does not play an important role in the computation of stiffness (K), with very low variations between using and not using G.
- It should be emphasized that due to the low number of samples used for each layout, the results of this work should be considered only in qualitative and not in quantitative terms. In a future work, the elaboration of the glulam timber using the technology of the finger joints as performed industrially will be considered.

## Figures and Tables

**Table 1:** Main mechanical properties of the tested samples. MoE_st_: static modulus of elasticity. MoE_FEM,G_: numerical modulus of elasticity using G. MoE_dyn,gt_: dynamic modulus of elasticity. MoE_c_: semi-anaytical modulus of elasticity. $\sigma_{max}$ : Maximum stress. PPi: Single-species pine. PPo: Single-species poplar. MPoPi: mixed poplar/pine. T^o^: Outer planks. T^i^: Inner planks.

Sample Name	Planks SC	MoE_st_ (MPa)	MoE_FEM,G_ (MPa)	MoE_dyn,gt_ (MPa)	MoE_c_ (MPa)	$\sigma_{max}\;(\mathrm{MPa})$
PPi1	T18	14,003	13,030	12,420	12,463	62.3
PPi2	T24	15,644	15,544	14,888	14,604	65.8
PPo1	T8	8703	8603	7689	7554	39.7
PPo2	T10	9098	8908	8699	8462	49.8
MPoPi1	T^o^24, T^i^8	16,162	15,021	11,612	13,328	67.4
MPoPi2	T^o^30, T^i^8	18,116	17,444	12,557	16,332	70.6

**Table 2:** Strength grading for the tested samples according to the different modulus of elasticity. MoE_st_: static modulus of elasticity. MoE_dyn,gt_: dynamic modulus of elasticity. MoE_c_: semi-anaytical modulus of elasticity. PPi: single-species pine. PPo: single-species poplar. MPoPi: mixed poplar/pine. T^o^: Outer planks. T^i^: Inner planks.

Sample Name	SC Planks	SC Design	SC (MoE_st_)	SC (MoE_dyn,gt_)	SC (MoE_c_)
PPi1	T18	GL28h	GL30h	GL26h	GL26h
PPi2	T24	GL32h	GL32h	GL32h	GL32h
PPo1	T8	-	GL20h	-	-
PPo2	T10	GL20h	GL20h	GL20h	GL20h
MPoPi1	T^o^24, T^i^8	-	GL32c	GL24c	GL30c
MPoPi2	T^o^30, T^i^8	-	GL32c	GL28c	GL32c

**Table 3:** Parallel Axes theorem results for the single-species pine PPi2 and mixed poplar/pine MPoPi1 samples. T^o^: Outer planks. T^i^: Inner planks. MoE_dyn,p_: dynamic modulus of elasticity of each plank. MoE_c_: semi-analytical dynamic modulus of elasticity.

Sample	SC Planks	MoE_dyn,p_ (MPa)	Contribution to the Total MoE_c_ (%)
PPi2	T^o^24	14,708	44.1
	T^i^24	14,932	6.4
	T^i^24	14,693	6.3
	T^o^24	14,441	43.3
MPoPi1	T^o^24	14,234	46.7
	T^i^8	7982	3.7
	T^i^8	7611	3.6
	T^o^24	14,002	46.0

**Table 4:** Variation in % of the elastic moduli respect to the static modulus MoE_st_. Comparison of the numerical and experimental moduli of elasticity. MoE_st_: static modulus of elasticity. MoE_FEM,G_: calibrated modulus of elasticity using G. MoE_dyn,gt_: dynamic modulus of elasticity. MoE_dyn,c_: semi-analytical modulus of elasticity. PPo: Single-species poplar. MPoPi: mixed poplar/pine.

Sample Name	Variation of MoE_FEM,G_ (%)	Variation of MoE_dyn,gt_ (%)	Variation of MoE_dyn,c_ (%)
PPi1	7.5	12.7	12.4
PPi2	0.6	5.1	6.0
PPo1	1.2	13.2	15.2
PPo2	2.1	4.6	7.5
MPoPi1	7.6	39.2	21.3
MPoPi2	3.8	44.3	10.9

**Table 5:** Comparison of experimental and numerical moduli of elasticity without considering the shear modulus and their variation in % respect to the static modulus MoE_st_. MoE_st_: static modulus of elasticity. MoE_st,g_: global static modulus of elasticity. MoE_FEM_: calibrated modulus of elasticity without using G.

Sample Name	MoE_st,g_ (MPa)	Variation of MoE_st,g_ (%)	MoE_FEM_ (MPa)	Variation MoE_FEM_ (%)
PPi1	11,474	22.0	11,000	27.3
PPi2	12,830	21.9	12,544	24.7
PPo1	7976	9.1	7503	16.0
PPo2	8322	9.3	8008	13.6
MPoPi1	11,955	35.2	11,260	43.5
MPoPi2	13,801	31.3	13,444	34.7

**Table 6:** Comparison of the shear moduli of elasticity. G_ex_: experimental shear modulus. G_FEM_: calibrated shear modulus used for numerical simulation.

Sample Name	G_ex_ (MPa)	G_FEM_ (MPa)
PPi1	300	300
PPi2	361	317
PPo1	451	401
PPo2	460	410
MPoPi1	217	229
MPoPi2	274	284

**Table 7:** Experimental and numerical stiffness used for the calibration of the FEM model. K_ex_: experimental stiffness. K_FEM,G_: calibrated numerical stiffness using G. K_FEM_: calibrated numerical stiffness without using G. Variation in % computed respect to K_ex._

Sample Name	K_ex_ (N/mm)	K_FEM,G_ (N/mm)	Variation of K_FEM,G_ (%)	K_FEM_ (N/mm)	Variation of K_FEM_ (%)
PPi1	760	761	0.1	765	0.5
PPi2	850	880	3.4	881	3.5
PPo1	529	548	3.5	552	1.3
PPo2	551	565	2.5	564	2.2
MPoPi1	792	805	1.6	783	1.2
MPoPi2	915	949	3.6	935	2.2

## Abbreviations

**Greek symbols:**	
σ_max_	Maximum normal stress in bending
δ_20,_δ_40_	Beam deflection at 20% and 40% of the maximum load
ΔL	Applied load increment
Δδ	Vertical displacement increment
ν	Poisson’s ratio
ρ_p_	Plank density
σ_y_	Yield stress
υ	Propagation velocity
**Symbols:**	
a	Distance between the load cell and nearest support
Ap	Cross-section of the sample
b	Section width
F_max_	Maximum applied force
G	Shear modulus
h	Section depth
Ic	Combined second moment of inertia about strong axis of the sample
Ip	Second moment of inertia about strong axis of the sample
K	Beam stiffness
L	Plank length/Beam length/Distance between supports
L_20,_L_40_	Applied load at 20% and 40% of the maximum load
M_max_	Maximum bending moment
W	Section modulus of the cross-section
**Acronyms:**	
3D-FEM	Three-dimensional finite element model
ART	Acoustic Resonance Testing
EWP	Engineered Wood Products
FAO	Food and Agriculture Organization
FEM	Finite element method
Gex	Experimental shear modulus
G_FEM_	Numerical shear modulus
HR	Room humidity
LVDT	Linear variable differential transformer
LVM	Longitudinal vibration method
MC	Reference moisture content (12%)
MC_p_	Measured moisture content
MoE_c_	Combined modulus of elasticity
MoE_dyn,gt_	Dynamic modulus of elasticity of the beam
MoE_dyn,p_	Dynamic modulus of elasticity of the plank
MoE_FEM_	Numerical modulus of elasticity without considering the shear modulus
MoE_FEM,G_	Numerical modulus of elasticity considering the shear modulus
MoE_st_	Static modulus of elasticity of the beam
MoE_st,g_	Global stati modulus of elasticity
MPoPi	Mixed poplar/pine
Ppi	Single-species pine
PPo	Single-species poplar
SC	Strength class
TSM	Transform section method
